# Effect of Sodium Selenate and Selenocystine on Savoy Cabbage Yield, Morphological and Biochemical Characteristics under *Chlorella* Supply

**DOI:** 10.3390/plants12051020

**Published:** 2023-02-23

**Authors:** Marina Antoshkina, Nadezhda Golubkina, Pavel Poluboyarinov, Liubov Skrypnik, Agnieszka Sekara, Alessio Tallarita, Gianluca Caruso

**Affiliations:** 1 Analytical Laboratory Department, Federal Scientific Vegetable Center, 143072 Moscow, Russia; 2 Medical Faculty, Department of General and Clinical Pharmacology, Penza State University, 440026 Penza, Russia; 3 Institute of Living Systems, Immanuel Kant Baltic Federal University, 236040 Kaliningrad, Russia; 4 Department of Horticulture, Faculty of Biotechnology and Horticulture, University of Agriculture, 31-120 Krakow, Poland; 5 Department of Agricultural Sciences, University of Naples Federico II, Portici, 80055 Naples, Italy

**Keywords:** *Brassica oleracea* L. var. sabauda, selenium biofortification, microalgae, production, head density, antioxidants

## Abstract

Biofortification of *Brassica oleracea* with selenium (Se) is highly valuable both for human Se status optimization and functional food production with direct anti-carcinogenic activity. To assess the effects of organic and inorganic Se supply for biofortifying *Brassica* representatives, foliar applications of sodium selenate and selenocystine (SeCys_2_) were performed on Savoy cabbage treated with the growth stimulator microalgae *Chlorella*. Compared to sodium selenate, SeCys_2_ exerted a stronger growth stimulation of heads (1.3 against 1.14 times) and an increase of leaf concentration of chlorophyll (1.56 against 1.2 times) and ascorbic acid (1.37 against 1.27 times). Head density was reduced by 1.22 times by foliar application of sodium selenate and by 1.58 times by SeCys_2_. Despite the greater growth stimulation effect of SeCys_2_, its application resulted in lower biofortification levels (2.9 times) compared to sodium selenate (11.6 times). Se concentration decreased according to the following sequence: leaves > roots > head. The antioxidant activity (AOA) was higher in water extracts compared to the ethanol ones in the heads, but the opposite trend was recorded in the leaves. *Chlorella* supply significantly increased the efficiency of biofortification with sodium selenate (by 1.57 times) but had no effect in the case of SeCys_2_ application. Positive correlations were found between leaf and head weight (r = 0.621); head weight and Se content under selenate supply (r = 0.897–0.954); leaf ascorbic acid and total yield (r = 0.559), and chlorophyll (r = +0.83–0.89). Significant varietal differences were recorded for all the parameters examined. The broad comparison performed between the effects of selenate and SeCys_2_ showed significant genetic differences as well as important peculiarities connected with the Se chemical form and its complex interaction with *Chlorella* treatment.

## 1. Introduction

The biofortification of agricultural crops with selenium (Se) is one of the most interesting methods to optimize human Se status [1]. Many years of experience regarding Se-enriched mineral fertilizers in Finland were accompanied by a significant decrease in population mortality due to cardiovascular diseases and cancer [2]. Indeed, this approach entails the production of functional food with high Se levels and enhanced the concentration of other natural antioxidants, valuable for health maintenance [3]. In biological systems, Se easily substitutes sulfur in organic compounds, indicating high biofortification prospects of Se-accumulating plants, such as *Allium* species and Brassicaceae members [4,5,6]. Anti-carcinogenic properties of methylated Se-containing amino acids and peptides [7,8] and Se-derivatives of Brassicaceae species’ glucosinolates [9,10] stimulated the development of Se biofortification methods, with highly efficient foliar supply [11]. Among *Brassica oleracea* sub-species, Se biofortification was achieved in broccoli [4,12], white and red cabbage [3,6], cauliflower [13,14], and kohlrabi [15,16]. Nevertheless, up-to-date peculiarities of *Brassica oleracea* plant biofortification with Se are still not clear. Among other members of this family, Savoy cabbage has never been reported to have been fortified with Se. 

Selenate, selenite, and to a lesser extent nano-Se are the most frequently utilized forms of Se for plant biofortification [3,8,12], whereas much less is known about the accumulation of organic Se forms. Banuelos et al. [4] demonstrated the efficiency of soil supplementation with Se-rich *Stanleya pinnata* to biofortify broccoli. Se-enriched peat has been used as a source of organic Se for cucumber, tomato, and lettuce production [17]. Seleno-amino acids can be applied to the soil via Se-amended organic manures [18]. Compared to selenate, SeMet provided higher biofortification levels in garlic and Indian mustard [19], whereas no differences between these Se derivatives were recorded in the biofortification of wheat, barley, and oats [20]. Previously, we had found that selenocystine (SeCys_2_) was more effective than selenate for biofortifying shallot plants in the presence of arbuscular mycorrhizal fungi (AMF), while the opposite phenomenon occurred with no AMF supply [21]. 

In nature, Se derivatives of amino acids are found in plant tissues. In this respect, SeMet has been detected in wheat and other cereals [22], while SeCys_2_ predominates in the Chinese Se-hyperaccumulator *Cardamine enshiensis* [23] as well as in *Astragalus bisulcatus* and *Stanleya pinnata* [24]. A significant amount of SeCys_2_ was also recorded in wheat grain [22]. The development of a cheap selenocystine synthesis by Poluboyarinov [25] provided an opportunity to widely use this compound to optimize human Se status, as food supplement, in premixes for domestic animals and poultry, and for fertilizer supplementation [26].

However, to date there are still extremely limited data regarding the mechanism of organic Se compounds utilization and assimilation by different plant species. 

Furthermore, the utilization of natural biostimulants for plant biofortification may open a new era of functional food production with high Se concentration excluding the risk of possible selenium toxicity during the crop growing [27]. In this regard, microalgae may be of special interest to stimulate plant growth and improve the tolerance to environmental stresses [28]. They can be used at least in two forms, i.e., fortified or not fortified with mineral elements [29]. The lack of experimental data about the interaction between microalgae and plant Se biofortification demonstrates the need for a detailed investigation of this topic.

The present research aimed to compare the biofortification efficiency of foliar applied organic and inorganic Se forms, in terms of growth, yield, quality, and antioxidant properties of Savoy cabbage plants treated with the microalgae *Chlorella*. 

## 2. Results and Discussion

### 2.1. Yield and Morphological Characteristics of Savoy Cabbage

Selenium biofortification is known to improve plant antioxidant status, membrane stability, photosynthesis, and mineral homeostasis, thus enhancing plant growth and development [30,31]. The data presented in Table 1 indicate a beneficial influence of organic and inorganic forms of Se on Savoy cabbage mean head weight and marketable yield, though the effect intensity differed between cultivars. Indeed, SeCys_2_ increased the mean head weight of cultivars Melissa and Vertu 1340 by 29.4 to 29.6% and marketable yield by 35.0 to 35.8%, while cv. Golubtsy demonstrated only a tendential increase of these parameters, with no statistically significant differences compared to the control plants.

Though *Chlorella* is known to improve plant nutrition and alleviate environmental stress [28], no beneficial effect of *Chlorella* application was recorded in the present work. *Chlorella* supplementation did not affect the mean head weight and marketable yield, but the increase of mean head weight and marketable yield as a result of joint Se + *Chlorella* supply compared to control plants exclusively reflected the beneficial effect of organic and inorganic Se forms (Table 1). Furthermore, all the treatments applied, i.e., Se and/or *Chlorella*, significantly increased the Savoy cabbage leaf weight, to a remarkable extent under joint selenate + *Chlorella* application. 

The growth-promoting effect of Se biofortification using low concentrations of Se has been previously recorded in many plant species [30,31], though cabbage biofortification with SeCys_2_ has been performed in the present research for the first time.

**Figure 1:**
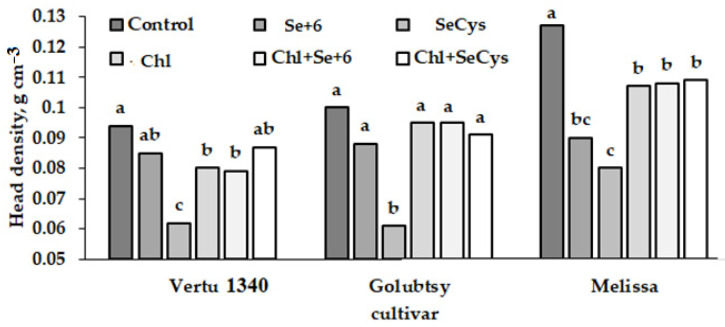
Head density of Savoy cabbage as affected by selenium biofortification and *Chlorella* application. Values with the same letters do not differ statistically according to the Duncan test at *p* < 0.05.

Taking into account the equation describing oblate spheroid volume and the head weight data related to the Savoy cabbage, we assessed the effects of single and joint application of different Se forms and microalgae *Chlorella* on the Savoy cabbage head density (Figure 1).

The data presented in Figure 1 reveal the phenomenon of a significant head density decrease as a result of *Chlorella*, inorganic and especially organic Se treatments, with the values decreasing under SeCys_2_ supply depending on the cultivar: by 1.52 (Vertu 1340) to 1.59 (Melissa) and 1.64 times (Golubtsy).

Interestingly, the joint sodium selenate and *Chlorella* supply did not significantly affect the head density compared to Se treatment, whereas the joint application of SeCys_2_ and *Chlorella* significantly enhanced the head density. It may be supposed that both Se forms delay the Savoy cabbage senescence, as was previously demonstrated in other plant species [30], via regulation of antioxidant enzymes’ activity, downregulation of ethylene production [32], and increasing chlorophyll content [33].

The results indicate the contrasting trends between head density and marketable yield (Table 1, Figure 1). In this respect, it may be inferred that though SeCys_2_ treatment significantly improved yield, at the same time it decreased the head density. In all cultivars, the density of control plant heads was either greater (cv. Melissa) or tendentially higher compared to the other treatments. Positive correlation coefficients were recorded between leaves and head weight (r = 0.621, *p* < 0.05); total and leaves weight (r = 0.625, *p* < 0.05) and total and head weight (r = 0.984, *p* < 0.001). In contrast, negative correlations were found between head density, total weight, and mean head weight (r = 0.571 to 0.580, *p* < 0.05).

### 2.2. Selenium Accumulation

**Figure 2:**
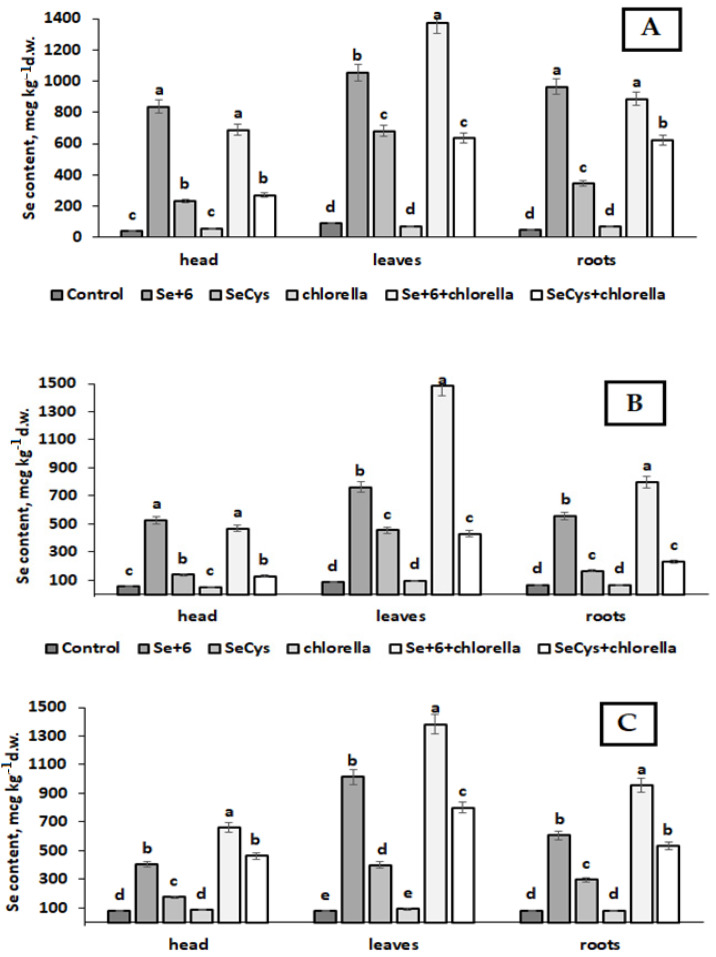
Selenium accumulation in Savoy cabbage as affected by Se and *Chlorella* supply. Cultivars: (**A**)—Vertu 1340; (**B**)—Golubtsy; (**C**)—Melissa. Values with the same letters do not differ statistically according to the Duncan test at *p* < 0.05.

The data of the present investigation indicate that Savoy cabbage Se biofortification provided significant accumulation of this microelement, with concentrations depending on Se chemical form and *Chlorella* supply (Figure 2). Previous investigations demonstrated that *Brassica oleracea* representatives are able to convert sodium selenate to selenomethionine [34]. Selenocystine is reportedly assimilated by plants via amino acid transporters, in contrast to inorganic selenate, which is absorbed through the sulfate pathway [35]. 

The comparison between selenate and SeCys_2_ in terms of biofortification efficiency unveils the greater increase of Se in leaves and the relatively low biofortification levels of plants subjected to SeCys_2_. Indeed, Se levels in Savoy cabbage leaves treated with sodium selenate ranged from 1026 to 1372 µg kg^−1^ d.w. (Vertu 1340), 667–1487 µg·kg^−1^ d.w. (Golubtsy), 1013 to 1383 µg·kg^−1^ d.w. (Melissa), whereas head Se content varied from 867 to 688 µg·kg^−1^ d.w. (Vertu 1340), 694 to 466 µg·kg^−1^ d.w. (Golubtsy), and 404 to 661 µg·kg^−1^ d.w. (Melissa). In contrast, much lower levels were recorded for the SeCys_2_ application: 235–269 µg·kg^−1^ d.w. (Vertu 1340), 138–128 µg·kg^−1^ d.w. (Golubtsy) and 176–464 µg·kg^−1^ d.w. (Melissa). A predominant Se accumulation in leaves was detected, compared to head, particularly significant under the joint selenate + *Chlorella* application. 

**Figure 3:**
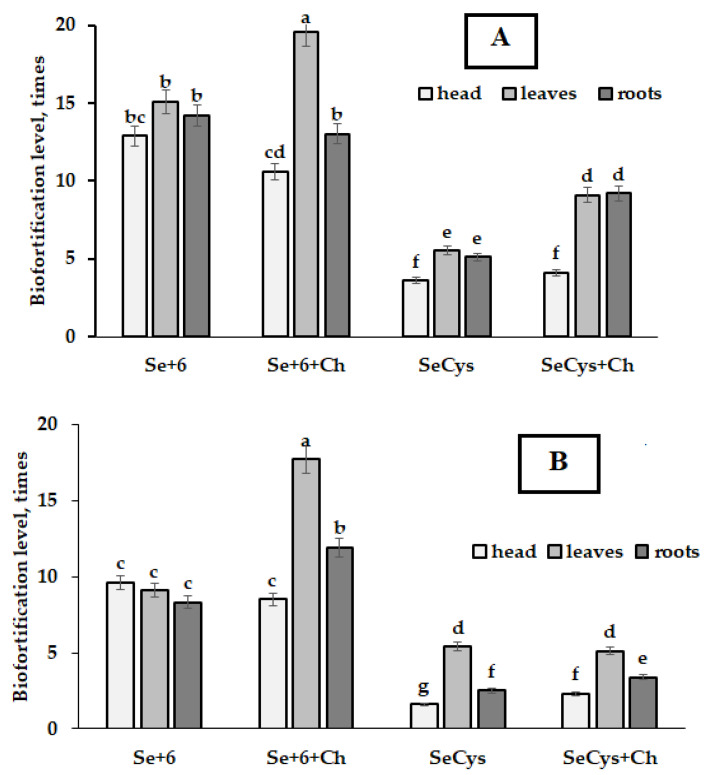

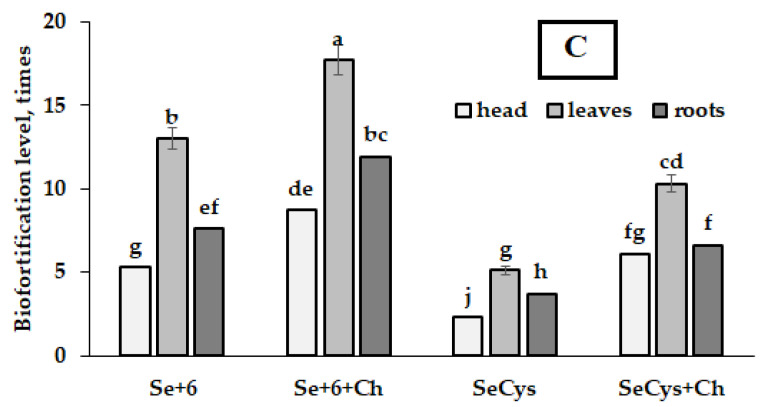
Selenium biofortification levels of the Savoy cabbage plant parts as affected by Se chemical form and *Chlorella* supply. Cultivars: (**A**)—Vertu 1340; (**B**)—Golubtsy; (**C**)—Melissa. Values with the same letters do not differ statistically according to Duncan test at *p* < 0.05.

As can be seen in Figure 2 and Figure 3, *Chlorella* supply showed complex effects. Indeed, it did not affect Se accumulation in control plants but significantly improved Se levels in case of joint selenate + *Chlorella* application. Selenocystine treatment under *Chlorella* supply elicited varietal differences: significant increase of Se level in Vertu 1340 roots, and in Melissa roots, head, and leaves. However, only in cv. Melissa, *Chlorella* demonstrated significant Se increase in all plant parts upon the joint applications of selenate + *Chlorella* and SeCys_2_ + *Chlorella* (Figure 2). 

The analysis of biofortification levels revealed the predominance of leaf Se accumulation, especially in case of joint selenate + *Chlorella* supply (Figure 3A–C). Much less significant effect or even its absence was recorded for roots and especially head biofortification levels. 

The presented data suggest that the foliar Se supply of Savoy cabbage resulted in predominant Se accumulation in leaves, with the highest concentration in sodium selenate treated plants, while SeCys_2_ led to much lower levels of Se biofortification. Figure 2 and Figure 3 show that *Chlorella* application may significantly increase organic and inorganic Se accumulation in leaves, head, and roots only in cultivar Melissa, with the predominance of leaf and root Se increase in cv. Vertu 1340, both under sodium selenate and SeCys_2_ supply, whereas in cv. Golubtsy, *Chlorella* supply was beneficial only to improve sodium selenate assimilation. 

Notably, microalgae reportedly improve plant nutrition [28] but their effect on Se accumulation has never been investigated. *Chlorella* ability to increase sodium selenate biofortification levels is of great importance to produce functional food with high content of this element. On the other hand, the complexity of *Chlorella*–SeCys_2_ interaction demonstrates the need for further investigations.

**Figure 4:**
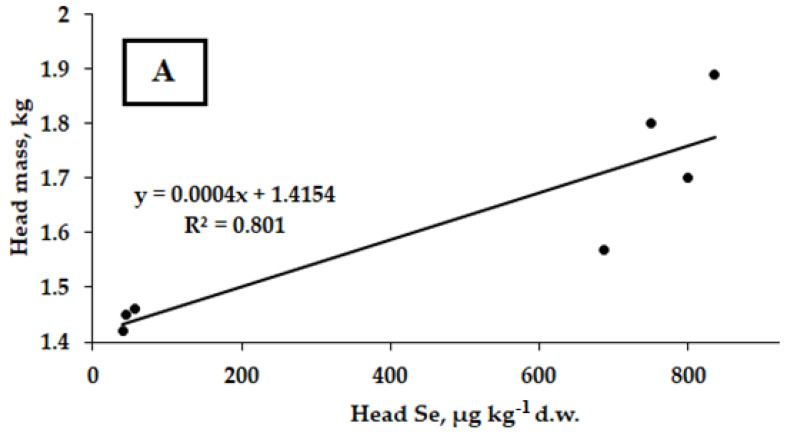

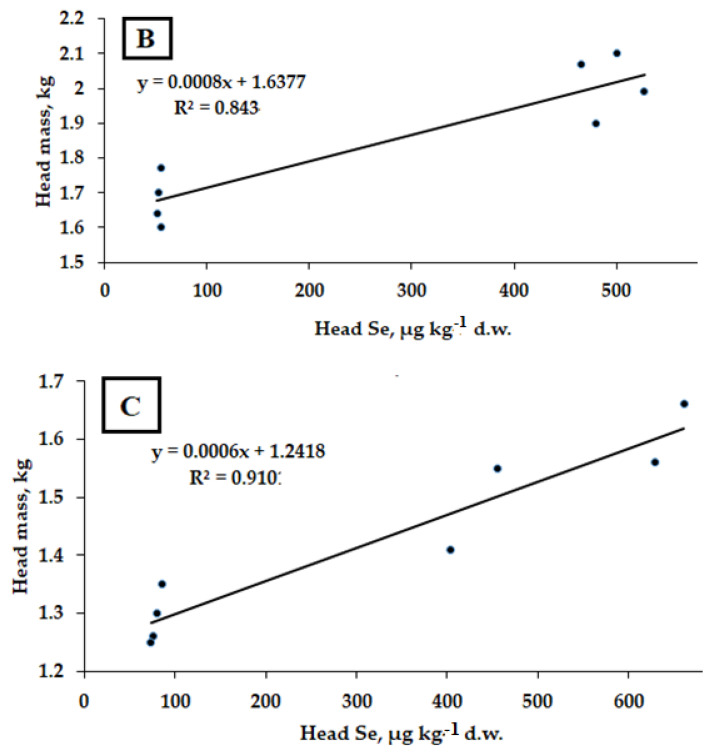
*Correlation* between head Se levels and weight of Savoy cabbage treated with sodium selenate and *Chlorella*. (**A**): Vertu 1430 (r = 0.897; *p* < 0.001); (**B**): Golubtsy (r = 0.918; *p* < 0.001); (**C**): Melissa (r = 0.954; *p* < 0.001).

Taking into account the beneficial effect of *Chlorella* supply only on selenate accumulation in Savoy cabbage, a relationship between head Se levels and weight has been evaluated based on the results obtained in 2021 and 2022 (Figure 4).

Though the SeCys_2_ treatment improved head weight more significantly than sodium selenate (Table 1), the determination of the appropriate correlation coefficients is exigent due to (i) lack of *Chlorella* beneficial effect on organic Se accumulation and (ii) significant varietal differences in plant response to Se/*Chlorella* supply, which excludes the possibility of calculating a single correlation coefficient for all three cultivars.

More remarkable beneficial effect of SeCys_2_ on the Savoy cabbage growth and development, compared to sodium selenate, is connected with the well-known growth stimulation effect of amino acids [36,37]. Among amino acids, Cys acts either as a chelating agent accelerating the absorption and transport of microelements within plants, or as an important component of plant enzymes. In this respect, it may be supposed that the highest efficiency of Cys/SeCys_2_ supply may be obtained in sulfur-accumulator plants, such as *Brassica* and *Allium* species. Indeed, recent investigations indicate that Cys significantly stimulates biologically active compounds synthesis in garlic, improving allicin content [38].

### 2.3. Antioxidants

The response of plant antioxidants to Se and *Chlorella* treatments was rather specific.

#### 2.3.1. Photosynthetic Pigments

At low concentrations, Se is known to stimulate the biosynthesis of photosynthetic pigments [30], which is directly connected with its growth promoting effect. The data presented in Table 2 indicate significant increase in chlorophyll and carotene content due to Se treatment either singly or in combination with *Chlorella* supply. It is important that SeCys_2_ resulted in a more remarkable increase of photosynthetic pigments accumulation compared to sodium selenate, both singly and in combination with *Chlorella*.

In this respect, the chlorophyll a/b ratio was the highest in plants treated with SeCys_2_, reaching 1.62–1.76 in cv. Vertu 1340, 1.25–1.27 in Golubtsy and 1.42 in Melissa. Cv. Golubtsy showed the lowest leaf levels of chlorophyll ‘a’ and ‘b’. 

*Chlorella vulgaris* is one of the most commercially available microalgae. Its utilization in agriculture is connected with its ability to provide simultaneously biofertilization, biostimulation and protection of plants against biotic and abiotic stresses [28]. *Chlorella* is known to improve cell metabolism, nucleic acid synthesis, photosynthesis, antioxidant status of plants, and water availability [39]. The results of the present work show that the beneficial effect of *Chlorella* supply is variety dependent. Indeed, the microalgae improved significantly chlorophyll ‘a’ and carotene content in Melissa leaves, only chlorophyll ‘a’ content in Vertu 1340, and had no effect on photosynthetic pigments accumulation in Golubtsy. In the present study, the lack of significant *Chlorella* effect on photosynthetic pigments accumulation and head weight may be connected with the low concentration applied, as 10-fold higher concentrations were shown to increase significantly yield and chlorophyll content in Swiss chard [40]. At the same time, it should be highlighted that significant biomass increase as a result of *Chlorella* supply was recorded only for the outer leaves of Savoy cabbage (Table 1).

**Figure 5:**
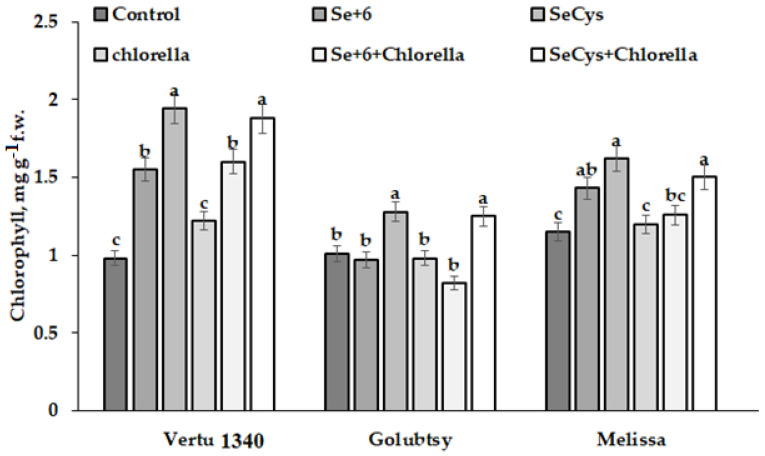
Chlorophyll accumulation in Savoy cabbage as affected by selenium biofortification and *Chlorella* supply. Values with the same letters do not differ statistically according to the Duncan test at *p* < 0.05.

A greater beneficial effect on photosynthetic pigments accumulation was recorded under Se application both singly or in combination with *Chlorella* (Figure 5). The data shown in Figure 5 indicate higher beneficial effect of SeCys_2_ on total chlorophyll content compared to inorganic form of Se, with the highest expression in Vertu 1340 leaves and the lowest in Golubtsy.

Furthermore, organic Se demonstrated a stronger beneficial effect on carotenoids accumulation in Savoy cabbage leaves (Table 2). Carotenoids have a major function to protect chlorophyll and the surrounding cells from light damage converting the excess excitation energy to heat [41]. The beneficial effect of Se on photosynthetic pigments accumulation is in accordance with the known increase of the photosystem II quantum efficiency in plants due to Se supply [42,43,44]. The increase of Savoy cabbage biomass under Se biofortification agrees with the mentioned results.

The comparison of variation coefficients (CV) for the morphological parameters tested (Table 1) and photosynthetic pigments accumulation (Table 2) indicated that, among the three cultivars examined, Vertu 1340 was the most sensitive to Se/*Chlorella* treatment effect on leaf weight and chlorophyll accumulation, the CV values reaching 20.7% and 25% respectively, while the cultivars Golubtsy and Melissa demonstrated much lower variations of the parameters tested.

#### 2.3.2. Ascorbic Acid

Improvement of the ascorbic acid biosynthesis due to Se application is directly connected with the intensity of photosynthesis. The data presented in Table 3 indicate the highest beneficial effect of Se supply on the ascorbic acid accumulation by cvs. Vertu 1340 and Golubtsy and no statistically significant changes of this parameter in cv. Melissa. The ascorbic acid levels in Golubtsy leaves increased under Se supply by 1.31–1.60 times and in heads by 1.25–1.48 times. The corresponding changes were recorded in Vertu 1340 leaves treated with sodium selenate, SeCys_2_, and a combination of SeCys_2_ + *Chlorella* (1.41–1.50 time). Significant changes of ascorbic acid content in Vertu 1340 were demonstrated only for plants treated with SeCys_2_ (1.28 times). In general, the present results indicate the low effect of *Chlorella* foliar supply on the ascorbic acid biosynthesis in Savoy cabbage.

**Figure 6:**
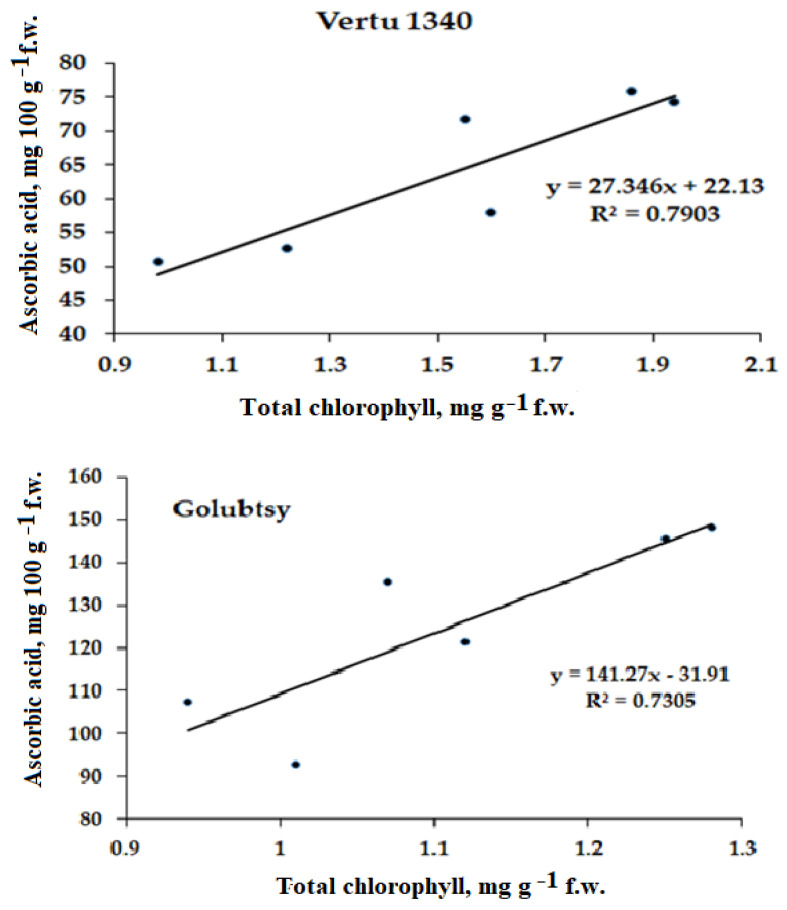

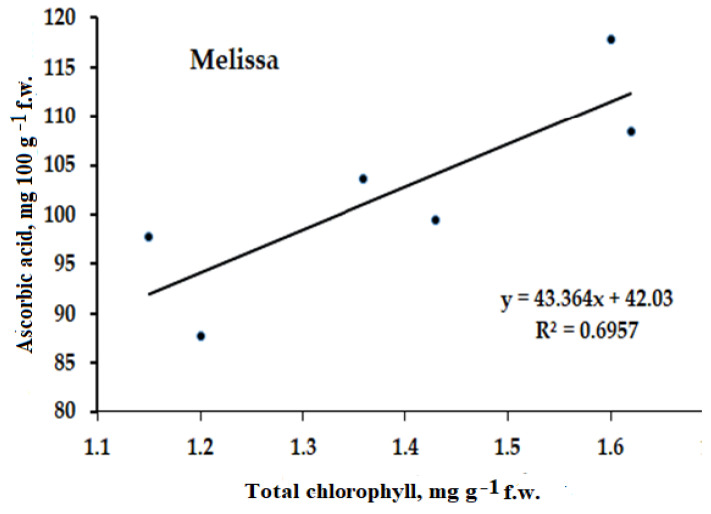
Correlation between ascorbic acid and chlorophyll content in the Savoy cabbage leaves. Vertu 1340: r = 0.89 (*p* < 0.005), Golubtsy: r = 0.85 (*p* < 0.01), Melissa: r = 0.83 (*p* < 0.01).

Ascorbic acid is an important cofactor for several enzymes involved in the synthesis of numerous secondary metabolites, including phytohormones, and a component in plant protection against environmental stresses [45]. In the present investigation, the highest content of ascorbic acid in leaves and head of Savoy cabbage corresponded to the highest leaves and head biomass (Table 1). The ascorbic acid increase due to Se supplementation was previously recorded in several leafy green vegetables [46,47,48], and enhanced plant Fe and Zn bioavailability [49]. The present data indicate that SeCys_2_/*Chlorella* supply increased the ascorbic acid content by 18–44% in Savoy cabbage head and by 21–60% in leaves, compared to control plants. Furthermore, the ascorbic acid biosynthesis in the Savoy cabbage leaves was positively correlated with the intensity of photosynthetic pigments accumulation (Figure 6). A similar relationship between ascorbic acid and photosynthetic pigments biosynthesis was previously found in canola exposed to salt stress [50]. Ascorbic acid demonstrates a significant role in the photosynthesis maintenance and in the protection of the photosynthetic apparatus against reactive oxygen species and photoinhibition [51].

According to the present results, the coefficient of correlation between the ascorbic acid content in leaves and the total Savoy cabbage biomass was 0.559 (*p* < 0.03).

#### 2.3.3. Total Antioxidant Activity (AOA)

Many plants contain more hydrophobic antioxidants than hydrophilic ones, which stimulates the utilization of 70% ethanol extraction during antioxidant activity determination [52].

**Figure 7:**
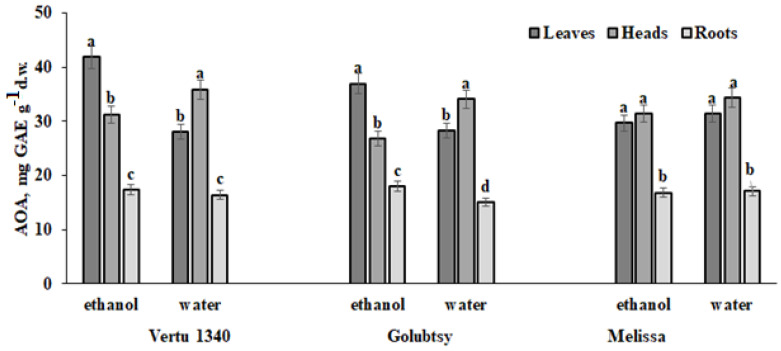
Mean AOA values for water and ethanol soluble antioxidants in leaves, head, and roots of the Savoy cabbage subjected to Se and *Chlorella* treatment without the data related to control plants. Within each cultivar, values with the same letters do not differ significantly according to the Duncan test at *p* < 0.05.

The present results indicate significant peculiarities in hydrophilic and hydrophobic antioxidant distribution in the leaves and head of the Savoy cabbage. Table 4 indicates the predominance of ethanol soluble antioxidants in leaves, and water soluble in the head. Moreover, significant differences between the levels of water and ethanol soluble antioxidants in leaves/ head regard predominantly the plants treated with Se and *Chlorella*, but not control plants. Significant accumulation levels of hydrophilic antioxidants in *Brassica oleracea* representatives were previously recorded by Podsedek et al. [53] in cabbage heads, but no information was given about the leaves and roots antioxidants distribution. The results of the present investigation demonstrated significant differences in hydrophobic and hydrophilic antioxidants content only in roots of control plants (Table 4, Figure 7). It may be supposed that Se/*Chlorella* treatments stimulate the mobility of root hydrophilic antioxidants and their transport to heads.

The fact that plant phenolics content (Table 5) did not differ significantly between the different experimental treatments suggests that AOA differences between Savoy cabbage leaves and head are not connected directly with the polyphenol profile of plants. The main phenolic acids in Savoy cabbage are gallic, sinapic, and chlorogenic, while quercetin and kaempferol were detected among flavonoids [54]. Heimler et al. [55] reported that, among different *Brassica oleracea* species, Savoy cabbage showed one of the highest levels of apigenin and luteolin [55]. 

Notably, the differences in antioxidant activity between water and 70% ethanol extracts are not connected either with polyphenols accumulation or ascorbic acid content due to the full degradation of the vitamin at 80 °C extraction for 1 h. In this respect, it may be supposed that water-soluble glucosinolates of the Savoy cabbage prevail in cabbage head compared to leaves. However, further investigations are needed to prove the above hypothesis. In 1979, VanEtten showed the predominance of oxidized glucosinolates forms in the Savoy cabbage head [56].

### 2.4. Dry Matter, Nitrates, Total Dissolved Solids (TDS), Carbohydrates 

No significant differences were detected between the different experimental treatments in terms of dry matter, nitrates, total dissolved solids, and carbohydrates content (Table 6). The leaves/head dry matter ratio changed from 1.61 to 1.74 and 1.74 (cultivars Vertu 1340, Golubtsy, and Melissa, respectively). The corresponding nitrate leaves/head ratio differed from 0.95 to 0.75 and to 0.80. The highest leaf dry matter and nitrate content were recorded in cultivar Golubtsy.

The total dissolved solids (TDS) reflect the content of water-soluble carbohydrates, minerals, nitrates, amino acids, and proteins. The high stability of TDS in cabbage leaves and heads in the present study is in accordance with the stability of nitrates and carbohydrates, both mono- and disaccharides (Table 6). In this respect, among the cultivars tested, Vertu 1340 was characterized by the highest sugar levels.

The active participation of Se in nitrogen metabolism via stimulation of the amino acid biosynthesis and increase of the nitrate reductase and glutamate synthase activity [46,57,58] did not arise in Savoy cabbage upon Se biofortification. Notably, Se–nitrogen interaction is species-specific and is significantly affected by plant hormonal status. In this respect, the lack of significant changes in the nitrate levels as a result of sodium selenate supplementation was recorded previously in kohlrabi [15] while intensive nitrate content decrease was found in wheat [59], sunflower [60], Indian mustard [46], lettuce [58,61,62], and potatoes [63]. The possibility of the hormonal factor participation in Se–nitrate reductase interaction was previously revealed in male and female forms of spinach plants [64]. It may be supposed that higher concentrations of sodium selenate and SeCys_2_ may cause significant changes in nitrates accumulation in Savoy cabbage, but this topic needs further investigation.

## 3. Material and Methods 

### 3.1. Growing Conditions and Experimental Protocol 

Research was conducted in 2021 and 2022, from April to October, at the experimental fields of the Federal Scientific Vegetable Center, Moscow region, Russia (55°39.51′ N, 37°12.23′ E), in a loam sod podzolic soil with the following characteristics: pH 6.2, 2.12% organic matter, 1.32 mg-eq 100 g^−1^ hydrolytic acidity, 18.5 mg kg^−1^ mineral nitrogen, 21.3 mg kg^−1^ ammonium nitrogen, sum of absorbed bases as much as 93.6%, 402 mg kg^−1^ mobile phosphorous, 198 mg kg^−1^ exchangeable potassium, 1 mg kg^−1^ S, 10.95 mg kg^−1^ Ca, 2.05 mg kg^−1^ Zn, 0.86 mg kg^−1^ B, 220 µg kg^−1^ d.w. Se, 7.65 mg kg^−1^ Ni, 0.22 mg kg^−1^ Cd, 1.6 mg kg^−1^ As, 12.85 mg kg^−1^ Pb.

The values of mean temperature and relative humidity during the crop cycles are presented in Table 7.

The sowing was performed on 27–29 April in multicell containers and the seedling planting on 2–4 June with a density of 2.9 plants per m^-2^ (50 × 70 cm). 

The experimental treatments, applied to three Savoy cabbage cultivars (Vertu 1340, Golubtsy, Melissa), were carried out according to the following scheme: (1) control (water foliar spray), (2) *Chlorella vulgaris* super concentrate suspension (‘Organic’ ltd, Tolyatti, Samara region, Russia) 35 mL per L, (3) sodium selenate solution, 26.4 mM (50 mg L^−1^), (4) SeCys_2_ solution, 26.4 mM (87 mg L^−1^), (5) sodium selenate + *Chlorella* (the same concentrations), and (6) SeCys_2_ + *Chlorella* (the same concentrations). A split plot design was used for the treatment distribution in the field, with three replicates and each experimental unit covering 10 m^2^. To exclude the interference of other factors, no fertilizers were applied during the experiment. The results were expressed as means of the two-year data.

The plants were sprayed with the appropriate solutions twice: at the stage of head formation (13 July) and 14 days later (4 August).

During the growing season, hoeing and manual weeding were carried out. A 4-fold treatment with insecticides BI-58 New (BASF Societas Europaea, Ludwichshafe, Germany) and Decis-Profi (Bayer Crop Science, Monheim, Germany) was carried out against herbivory.

Cabbage harvesting was carried out on 6–8 October.

### 3.2. Sample Preparation 

After harvesting and removal of soil particles from roots, leaves, heads, and roots of at least 10 plants were separated and homogenized. Fresh leaves and heads homogenates were used for the determination of nitrates, ascorbic acid, and photosynthetic pigments. The remaining parts of plants were dried at 70 °C to constant weight and homogenized, and the resulting powders were used for the determination of the total polyphenols content (TP), total antioxidant activity (AOA), and lipids content.

### 3.3. Head Density 

Savoy cabbage head density was determined using head mass and head volume, calculated according to [65] using the formula of spheroid volume:

V = 4/3 πD^2^H,

where V—head volume, cm^3^;

D—head diameter, cm;

H—head height cm.

### 3.4. Dry Matter 

The dry matter was assessed gravimetrically by drying the samples in an oven at 70 °C until constant weight. 

### 3.5. Nitrates

Nitrates were assessed using ion-selective electrode with ionomer Expert-001 (Econix Inc., Moscow, Russia) according to [66]. 

### 3.6. Ascorbic Acid 

The ascorbic acid content was determined by visual titration of leaf and head extracts in 3% trichloracetic acid with sodium 2.6-dichlorophenol indophenolate solution (Tillman’s reagent) [67]. Roots were not taken into consideration due to low ascorbic acid content. 

### 3.7. Photosynthetic Pigments 

Photosynthetic pigments were measured using 96% ethanolic extracts of Savoy cabbage leaves according to Lichtenthaler [68]. 

### 3.8. Total Polyphenols (TP) 

Total polyphenols were determined in 70% ethanol extracts of dried samples using the Folin–Ciocâlteu colorimetric method as previously described [69]. One gram of dry homogenates was extracted with 20 mL of 70% ethanol/water at 80 °C for 1 h. The mixture was cooled down and quantitatively transferred to a volumetric flask, and the volume was adjusted to 25 mL. The mixture was filtered through filter paper, and 1 mL of the resulting solution was transferred to a 25 mL volumetric flask, to which 2.5 mL of saturated Na_2_CO_3_ solution and 0.25 mL of diluted (1:1) Folin–Ciocâlteu reagent were added. The volume was brought to 25 mL with distilled water. One hour later the solutions were analyzed through a spectrophotometer (Unico 2804 UV, Suite E Dayton, NJ, USA), and the concentration of polyphenols was calculated according to the absorption of the reaction mixture at 730 nm. As an external standard, 0.02% gallic acid was used. The results were expressed as mg of gallic acid equivalent per g of dry weight (mg GAE g^−1^ d.w). 

### 3.9. Antioxidant Activity (AOA) 

The antioxidant activity of cabbage roots, heads, and leaves was assessed on (a) 70% ethanolic extracts of dry samples, and (b) water extracts, using a redox titration method [69]. The values were expressed in mg gallic acid equivalents (mg GAE g^−1^ d.w.).

### 3.10. Sugars 

Monosaccharides were determined using the ferricyanide colorimetric method, based on the reaction of monosaccharides with potassium ferricyanide [70]. Total sugars were analogically determined after acidic hydrolysis of water extracts with 20% hydrochloric acid. Fructose was used as an external standard. The results were expressed in% per dry weight.

### 3.11. Selenium 

Selenium was analyzed using the fluorometric method previously described for tissues and biological fluids [71]. Dried homogenized samples were digested via heating with a mixture of nitric–perchloric acids, subsequent reduction of selenate (Se^+6^) to selenite (Se^+4^) with a solution of 6 N HCl, and the formation of a complex between Se^+4^ and 2,3-diaminonaphtalene. Calculation of the Se concentration was achieved by recording the piazoselenol fluorescence value in hexane at 519 nm λ emission and 376 nm λ excitation. Each determination was performed in triplicate. The precision of the results was verified using a reference standard-lyophilized mitsuba stem in each determination with a Se concentration of 1865 µg·Kg^−1^ (Federal Scientific Vegetable Center). The results are expressed in µg kg^−1^ d.w.

### 3.12. Statistical Analysis 

Data were processed by analysis of variance, and mean separations were performed through the Duncan’s multiple range test, with reference to 0.05 probability level, using SPSS software version 21 (Armonk, NY, USA).

## 4. Conclusions

The results of the present research showed that sodium selenate had a greater biofortification effect on Savoy cabbage, compared to SeCys_2_, with the predominance of Se accumulation in leaves, though the growth stimulation effect was higher under organic Se supply which also provided a higher accumulation of photosynthetic pigments. The efficiency of sodium selenate biofortification may be increased with *Chlorella* foliar supply. Se treatment significantly decreased head density, but improved leaves yield. The beneficial effect of Se on plant yield and leaf AOA reveals the good prospects of Se biofortification in Savoy cabbage. 

## Figures and Tables

**Table 1:** Morphological characteristics and yield of Savoy cabbage biofortified by different forms of Se under *Chlorella* application.

Treatment	Head Diameter (cm)	Head Height (cm)	Mean Head Weight (kg)	Leaves Weight (kg)	Yield (kg)		
					Marketable	Non- Marketable	Total
Vertu 1340							
Control	18.3 a	10.8 c	1.42 b	0.69 c	34.6 c	5.2 a	39.8 b
Se^+6^	19.0 a	12.2 bc	1.57 ab	0.91 b	39.2 bc	4.8 a	44.0 b
SeCys_2_	21.2 a	15.8 a	1.84 a	1.19 a	47.0 ab	4.5 a	51.5 a
*Chlorella*	19.1 a	11.9 bc	1.46 b	1.08 ab	35.5 c	5.4 a	40.9 b
Se^+6^+ *Ch*	20.6 a	13.4 ab	1.89 a	1.28 a	48.3 a	4.6 a	52.9 a
SeCys_2_+ *Ch*	18.5 a	11.5 bc	1.44 b	1.21 a	35.6 c	4.7 a	40.3 b
M ± SD	10.5 ± 1.2	12.6 ± 1.8	1.60 ± 0.21	1.06 ± 0.22	40.4 ± 6.1	4.9 ± 0.4	45.3 ± 5.9
CV (%)	11.3	14.2	13.1	20.7	15.1	8.2	13.1
Golubtsy							
Control	19.3 b	11.6 a	1.77 ab	0.96 c	44.7 b	4.9 a	49.6 ab
Se^+6^	20.8 ab	12.5 a	1.99 ab	1.06 bc	51.1 ab	4.6 a	55.7 ab
SeCys_2_	24.7 a	13.8 a	2.16 a	1.38 ab	56.0 a	4.5 a	60.5 a
*Chlorella*	18.6 b	11.9 a	1.64 b	1.27 ab	41.1 b	4.8 a	45.9 b
Se^+6^+ *Ch*	20.1 ab	12.9 a	2.07 a	1.49 a	53.5 a	4.5 a	58.0 a
SeCys_2_+ *Ch*	19.7 b	12.4 a	1.84 a	1.19 b	46.8 b	4.7 a	51.5 ab
M ± SD	20.5 ± 2.2	12.5 ± 0.8	1.91 ± 0.20	1.22 ± 0.20	48.9 ± 5.6	4.5 ± 0.2	53.5 ± 5.5
CV (%)	10.6	6.2	10.5	16.4	11.5	4.4	10.3
Melissa							
Control	15.0 b	10.5 b	1.26 c	0.81 c	30.3 b	5.0 a	35.3 b
Se^+6^	17.1 ab	12.8 ab	1.41 abc	1.05 b	34.8 ab	4.7 a	39.5 ab
SeCys_2_	19.2 a	13.2 a	1.63 ab	1.23 a	40.9 a	4.7 a	45.6 a
*Chlorella*	15.8 ab	12.1 ab	1.35 bc	1.09 ab	32.9 b	4.9 a	37.8 b
Se^+6^+ *Ch*	16.9 ab	12.9 a	1.66 a	1.28 a	42.0 a	4.5 a	46.5 a
SeCys_2_+ *Ch*	16.2 ab	11.9 ab	1.43 abc	1.14 ab	35.3 ab	4.7 a	40.0 ab
M ± SD	16.7 ± 1.4	12.2 ± 1.0	1.50 ± 0.10	1.10 ± 0.17	36.0 ± 4.6	4.8 ± 0.1	40.8 ± 4.4
CV (%)	8.4	8.2	6.7	15.4	12.7	2.1	10.8

*Ch*: *Chlorella*. Within each cultivar and column, values with the same letters do not differ statistically according to the Duncan test at *p* < 0.05.

**Table 2:** Effect of Se biofortification under *Chlorella* supply on photosynthetic pigments accumulation in Savoy cabbage leaves (mg g^−1^).

	Chl a	Chl b	Car	Chl a/b	Chl/car
Vertu 1340					
Control	0.59 c	0.39 c	0.08 c	1.51 c	12.25 a
Se^+6^	1.01 ab	0.54 b	0.13 b	1.87 ab	11.92 a
SeCys_2_	1.2 a	0.74 a	0.17 a	1.62 bc	11.41 a
*Chlorella*	0.8 b	0.42 c	0.10 c	1.90 a	12.20 a
Se^+6^+ *Chlorella*	1.02 ab	0.58 b	0.15 ab	1.76 abc	10.67 a
SeCys_2_+ *Chlorella*	1.2 a	0.68 a	0.16 a	1.76 abc	11.75 a
M ± SD	0.97 ± 0.24	0.56 ± 0.14	0.13 ± 0.03	1.74 ± 0.15	11.77 ± 0.59
CV, %	24.7	25.0	23.1	8.6	5.0
Golubtsy cv.					
Control	0.53 b	0.48 ab	0.10 b	1.10 a	10.1 ab
Se^+6^	0.52 b	0.55 bc	0.10 b	1.16 a	9.70 ab
SeCys_2_	0.71 a	0.57 a	0.15 a	1.25 a	8.53 b
*Chlorella*	0.47 b	0.47 b	0.08 b	1.00 a	11.75 a
Se^+6^+ *Chlorella*	0.53 b	0.59 c	0.09 b	1.10 a	9.11 ab
SeCys_2_+ *Chlorella*	0.70 a	0.55 a	0.13 a	1.27 a	9.62 ab
M ± SD	0.56 ± 0.10	0.48 ± 0.05	0.11 ± 0.02	1.15 ± 0.10	9.45 ± 1.10
CV, %	17.8	10.3	18.2	8.7	11.6
Melissa cv.					
Control	0.57 c	0.58 b	0.10 c	1.00 b	11.50 a
Se^+6^	0.82 ab	0.61 ab	0.12 bc	1.34 a	11.92 a
SeCys_2_	0.95 a	0.67 a	0.18 a	1.42 a	9.00 b
*Chlorella*	0.70 b	0.50 b	0.14 b	1.40 a	8.57 b
Se^+6^+ *Chlorella*	0.65 bc	0.71 a	0.09 c	1.07 b	14.00 a
SeCys_2_+ *Chlorella*	0.88 a	0.72 a	0.17 a	1.42 a	8.82 b
M ± SD	0.76 ± 0.12	0.59 ± 0.04	0.13 ± 0.03	1.28 ± 0.19	10.38 ± 2.19
CV (%)	15.8	6.7	22.5	14.8	21.1

Within each column, values with the same letters do not differ statistically according to the Duncan test at *p* < 0.05.

**Table 3:** Ascorbic acid accumulation in leaves and heads of Savoy cabbage under selenium and *Chlorella* supply (mg 100 g^−1^ f.w.).

	Vertu 1340		Golubsty		Melissa	
	Leaves	Head	Leaves	Head	Leaves	Head
Control	50.7 b	42.5 b	92.8 d	46.5 c	97.7 a	50.5 ab
Se^+6^	71.7 a	47.2 ab	135.5 ab	66.4 a	99.4 a	51.1 ab
SeCys_2_	74.2 a	54.5 a	148.3 a	67.1 a	108.4 a	59.9 a
*Chlorella*	52.7 b	43.7 b	107.4 cd	50.2 bc	87.7 a	46.9 b
Se^+6^+ *Chlorella*	57.9 b	46.2 ab	121.3 bc	58.0 ab	103.7 a	57.6 a
SeCys_2_+ *Chlorella*	75.8 a	50.0 ab	145.5 a	66.6 a	117.8 a	59.3 a
M ± SD	63.8 ± 11.4	45.7 ± 7.1	125.1 ± 22.1	59.1 ± 9.1	102.5 ± 10.2	54.2 ± 5.4
CV (%)	17.9	15.5	17.7	15.4	10.0	10.0

Within each column, values with the same letters do not differ statistically according to the Duncan test at *p* < 0.05.

**Table 4:** Hydrophobic and hydrophilic extract antioxidant activity of the Savoy cabbage subjected to Se and *Chlorella* treatment (mg GAE g^−1^ d.w.).

	Leaves		Heads		Roots	
	Ethanol	Water	Ethanol	Water	Ethanol	Water
Vertu						
control	40.4 a	36.5 a	37.1 a	37.7 a	16 b	21.1 a
Se^+6^	44.4 a	25.9 c	30.2 b	35.6 a	14.9 b	15.8 b
SeCys_2_	41.4 a	25.1 c	29 b	37.6 a	17.9 ab	16.9 ab
*Chlorella*	37.1 a	30.3 abc	30.5 b	35.5 a	16.5 b	16.3 b
Se^+6^+ *Ch*	46.3 a	27 bc	34.3 ab	36 a	18.2 a	16.3 b
SeCys_2_+ *Ch*	40.5 a	32.3 ab	32.6 ab	34.6 a	19.5 a	16.8 b
M ± SD	41.7 ± 3.3 a	29.5 ± 4.4 b	32.3 ± 3.0 a	36.2 ± 1.2 a	17.2 ± 1.7 a	16.5 ± 0.5 a
Golubtsy						
control	29.6 b	31.7 a	27.4 a	36.2 ac	16.5 b	24.4 a
Se^+6^	37.2 a	26.9 a	24.5 a	42.2 a	18.2 b	15.6 bc
SeCys_2_	39.5 a	26.1 a	24.7 a	33.3 bc	17.9 b	14.5 c
*Chlorella*	34.8 ab	26.6 a	28.1 a	32.1 b	17.5 b	14.2 c
Se^+6^+ *Ch*	35.3 a	29.8 a	26.4 a	32.1 b	17.5 b	15.8 bc
SeCys_2_+ *Ch*	38.3 a	31.9 a	30.6 a	30.6 b	18.7 b	15.2 c
M ± SD	35.8 ± 3.5 a	28.8 ± 2.6 b	27.0 ± 2.3 b	34.4 ± 4.2 a	17.7 ± 0.7 a	16.6 ± 3.9 a
Melissa						
control	29.2 b	29 a	30.3 a	27.2 c	15.6 b	23.7 a
Se^+6^	36 a	33 a	28.5 a	36.4 ab	17.4 b	16.8 b
SeCys_2_	35 a	27.1 a	32 a	29.5 b	17.7 b	17.1 b
*Chlorella*	34.6 a	30.7 a	28.7 a	32 bc	15.6 b	16.0 b
Se^+6^+ *Ch*	39.2 a	30.7 a	32 a	34 ab	16.7 b	17.6 b
SeCys_2_+ *Ch*	39.3 a	27.1 a	35.8 a	39.9 a	16.5 b	18.0 b
M ± SD	35.6 ± 3.7 a	29.6 ± 2.3 b	31.2 ± 2.7 a	33.2 ± 4.6 a	16.6 ± 0.9 a	18.2 ± 2.8 a

*Ch*: *Chlorella*. Within each cultivar and column, values with the same letters do not differ significantly according to the Duncan test at *p* < 0.05.

**Table 5:** Mean values of polyphenol content in ethanol and water extracts of Savoy cabbage leaves, head, and roots (mg GAEg^−1^ d.w.).

Cultivar	Leaves		Head		Roots	
	70% Ethanol	Water	70% Ethanol	Water	70% Ethanol	Water
Vertu 1340	33.6 ± 1.7 ab	17.1 ± 0.7	30.7 ± 2.3 a	21.9 ± 1.8	11.4 ± 1.0 a	9.0 ± 0.6
CV (%)	5.1	4.1	7.5	8.2	8.8	6.7
Golubtsy	31.7 ± 0.8 b	17.0 ± 0.5	24.0 ± 1.4 b	21.3 ± 2.0	9.5 ± 0.7 ab	8.7 ± 0.8
CV (%)	2.5	2.9	5.8	9.4	7.4	9.2
Melissa	37.3 ± 2.3 a	17.3 ± 0.6	29.5 ± 1.7 a	21.5 ± 1.8	9.2 ± 0. 6 b	10.4 ± 0.8
CV (%)	6.2		5.8		6.5	7.7

Within each column, values with the same letters do not differ statistically according to the Duncan test at *p* < 0.05.

**Table 6:** Mean values of dry matter, nitrates, total dissolved solids (TDS), and carbohydrates in Savoy cabbage plants of control and fortified with Se under *Chlorella* supply.

Treatment	Vertu 1340		Golubtsy		Melissa	
	Leaves	Heads	Leaves	Heads	Leaves	Heads
Dry matter (%)	14.0 ± 0.46 b	8.68 ± 0.56 c	17.7 ± 0.66 a	10.2 ± 1.02 c	18.4 ± 0.22 a	10.6 ± 0.62 c
CV (%)	3.3	6.5	3.7	10.0	1.2	5.8
Nitrates, mg g^−1^ d.w.	3.6 ± 0.1 b	3.8 ± 0.3 b	3.8 ± 0.2 b	5.1 ± 0.6 a	3.7 ± 0.4 b	4.6 ± 0.3 a
CV (%)	3.39 ab	8.66	7.24	13.5	11.3	14.1
TDS, mg g^−1^ d.w.	89.3 ± 5.6 a	64.1 ± 2.0 b	92.6 ± 2.5 a	66.6 ± 2.4 b	93.3 ± 2.2 a	63.1 ± 1.7 b
CV (%)	6.22	3.19	2.69	3.61	2.38	2.71
Monosaccharides (% d.w.)	traces	47.62 + 4.67 a	traces	36.88 + 2.07 b	traces	33.98 + 2.00 b
CV (%)	-	9.81	-	5.61	-	5.90
Total sugar (% d.w.)	Traces	50.48 + 4.53 a	traces	39.25 + 1.41 b	traces	41.63 + 3.10 b
CV (%)	-	8.97	-	3.59	-	7.45

TDS: total dissolved solids. Along each line, values with the same letters do not differ statistically according to the Duncan test at *p* < 0.05.

**Table 7:** Monthly temperature and precipitation in 2021 and 2022.

Month	Temperature (°C)		Precipitation (mm)	
	2021	2022	2021	2022
May	13.8	10.0	81	55.5
June	21.8	18.6	20	24.6
July	22.0	20.2	38	66.1
August	19.4	22.3	36	13.7
September	9.1	9.6	58	125.7

## Data Availability

Not applicable.
